# Clinical and Molecular Epidemiology of an Emerging Panton-Valentine Leukocidin-Positive ST5 Methicillin-Resistant Staphylococcus aureus Clone in Northern Australia

**DOI:** 10.1128/mSphere.00651-20

**Published:** 2021-02-10

**Authors:** Sarah L. McGuinness, Deborah C. Holt, Tegan M. Harris, Connor Wright, Rob Baird, Phillip M. Giffard, Asha C. Bowen, Steven Y. C. Tong

**Affiliations:** a Department of Infectious Diseases, Royal Darwin Hospital, Darwin, Northern Territory, Australia; b Department of Epidemiology and Preventive Medicine, School of Public Health and Preventive Medicine, Monash University, Melbourne, Victoria, Australia; c Department of Infectious Diseases, The Alfred Hospital and Monash University, Melbourne, Victoria, Australia; d Global and Tropical Health Division, Menzies School of Health Research, Charles Darwin University, Darwin, Northern Territory, Australia; e Microbiology, Territory Pathology, Royal Darwin Hospital, Darwin, Northern Territory, Australia; f College of Health and Human Sciences, Charles Darwin University, Darwin, Northern Territory, Australia; g Perth Children’s Hospital, Perth, Western Australia, Australia; h Wesfarmers Centre for Vaccines and Infectious Diseases, Telethon Kids Institute, University of Western Australia, Perth, Western Australia, Australia; i School of Paediatrics and Child Health, University of Western Australia, Perth, Western Australia, Australia; j Victorian Infectious Disease Service, The Royal Melbourne Hospital, Parkville, Victoria, Australia; k Doherty Department, University of Melbourne, at the Peter Doherty Institute for Infection and Immunity, Victoria, Australia; University of Nebraska Medical Center

**Keywords:** *Staphylococcus aureus*, epidemiology, genomics, methicillin resistance, susceptibility testing, trimethoprim

## Abstract

Recently, we identified a Staphylococcus aureus sequence type 5 (ST5) clone in northern Australia with discrepant trimethoprim-sulfamethoxazole (SXT) susceptibility results. We aimed to identify isolates of this clone using Vitek 2 SXT resistance as a proxy and to compare its epidemiology with those of other circulating S. aureus strains. We collated Vitek 2 susceptibility data for S. aureus isolates collected through our laboratory and conducted a prospective, case-control study comparing clinical, microbiological, epidemiological, and genomic data for subsets of isolates reported as SXT resistant (cases) and SXT susceptible (controls) by Vitek 2. While overall SXT resistance rates remained relatively stable from 2011 to 2018 among 27,721 S. aureus isolates, non-multidrug-resistant methicillin-resistant S. aureus (MRSA) strains almost completely replaced multidrug-resistant MRSA strains as the predominant SXT-resistant MRSA phenotype. Demographic and clinical features of 51 case-control pairs were similar, but genotyping revealed stark differences: clonal complex 5 (CC5) MRSA predominated among SXT-resistant cases (34/51 [67%]), while CC93 MRSA predominated among susceptible controls (26/51 [51%]). All CC5 isolates were an ST5 clonal lineage that possessed the trimethoprim resistance gene *dfrG* within SCC*mec* IVo; all were SXT susceptible by Etest. The replacement of Vitek 2 reported SXT-resistant multidrug-resistant MRSA by non-multidrug-resistant MRSA appears related to the emergence of an ST5-MRSA-SCC*mec* IVo clone that is SXT susceptible by Etest and causes clinical disease similar to that caused by ST93-MRSA-SCC*mec* IVa. Reliance on Vitek 2 SXT reporting may lead to unnecessary restriction of effective oral treatment options for S. aureus infections. Whether the presence of *dfrG* within SCC*mec* IVo provides a selective advantage at the population level is currently unclear.

**IMPORTANCE** Staphylococcus aureus is an important human pathogen that causes a wide range of clinical infections. In the past 2 decades, an epidemic of community-associated skin and soft tissue infections has been driven by S. aureus strains with specific virulence factors and resistance to beta-lactam antibiotics. Recently, an S. aureus strain with discrepant antimicrobial susceptibility testing results has emerged in northern Australia. This ST5-MRSA-SCC*mec* IVo clone is reported as resistant to trimethoprim-sulfamethoxazole by Vitek 2 but susceptible by phenotypic methods. ST5-MRSA-SCC*mec* IVo is now the second most common community-associated MRSA clone in parts of Australia and causes a spectrum of clinical disease similar to that caused by the virulent ST93-MRSA lineage. Whole-genome sequence analysis demonstrates that ST5-MRSA-SCC*mec*IVo is causing a clonal outbreak across a large geographical region. Although phenotypic testing suggests *in vitro* susceptibility to trimethoprim-sulfamethoxazole, it is unclear at this stage whether the presence of *dfrG* within SCC*mec* IVo provides a selective advantage at the population level.

## INTRODUCTION

Staphylococcus aureus is a leading cause of skin and soft tissue infections (SSTIs), osteoarticular infections, bacteremia, infective endocarditis, and device-related infections worldwide. These infections are associated with considerable morbidity and mortality (1, 2). In the Western Pacific region (which includes Australia), there is a strikingly disproportionate burden of S. aureus disease in Indigenous communities (2, 3).

S. aureus is well recognized for its ability to develop antibiotic resistance: methicillin-resistant S. aureus (MRSA) rapidly appeared in hospitals after the introduction of methicillin and has emerged as a widespread cause of community infections (4, 5). Over the past 2 decades, an epidemic of SSTIs has arisen in community settings, principally driven by MRSA clones with specific virulence factors (2–4, 6). In North America, USA300 is overwhelmingly dominant, whereas the virulent sequence type 93 (ST93) MRSA clone (Queensland clone) has emerged as the leader in Australia (2, 7, 8). These community MRSA clones typically have a non-multidrug-resistant MRSA (nmMRSA) antimicrobial susceptibility profile, and non-beta-lactam agents are used to treat these infections (5).

Trimethoprim-sulfamethoxazole (SXT) is a broad-spectrum, combination sulfonamide antibiotic available in oral and intravenous formulations (9). SXT has activity against many community MRSA clones and is increasingly being recommended for the treatment of SSTIs in adults and children (10, 11). Following a recent randomized controlled trial (RCT) in which short-course SXT was demonstrated to be noninferior to intramuscular benzathine penicillin G in the treatment of impetigo (12), SXT has been recommended for (13) and adopted into (11, 14, 15) treatment guidelines. The emergence of SXT resistance in S. aureus would therefore have significant implications for prescribing guidelines and practice.

A recent 20-year (1993 to 2012) study of community S. aureus isolates in the Northern Territory (NT) of Australia demonstrated an increase in SXT resistance from 2010 to 2012, albeit at low rates (<2%) (3). In investigating presumed SXT-resistant strains recovered during the impetigo treatment RCT (12), a surprising discrepancy was found in antimicrobial susceptibility testing results for SXT as determined by different methodologies among 19 isolates from 8 of 508 children in the trial (16). The discrepant isolates tested resistant to SXT by Vitek 2 but susceptible by Etest (with further confirmation of SXT susceptibility with broth microdilution for 2 isolates). These isolates were a clonal Panton-Valentine leukocidin (PVL)-positive ST5-MRSA lineage (16). Notably, the emergence of a trimethoprim-resistant PVL-positive ST5-MRSA clone (WA121 MRSA) in the neighboring state of Western Australia (WA) has also been observed (17).

We therefore aimed to identify further isolates of this clone among infections in individuals presenting to a tertiary care center, using SXT resistance by Vitek 2 as a proxy for the clone. We had a particular focus on providing a more detailed clinical and molecular description of the lineage in comparison to other circulating S. aureus strains.

## RESULTS

### Changes in S. aureus resistance over time.

**FIG 1:**
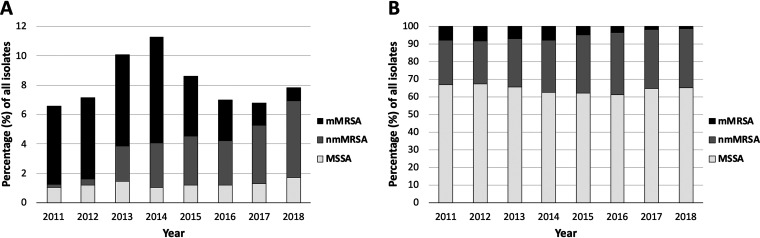
Proportion of 27,721 S. aureus isolates reported as SXT resistant by Vitek 2 by year (2011 to 2018), stratified by type (A) and proportion of 27,721 *S. aureus* isolates reported as MSSA, nmMRSA, and mMRSA by year (2011 to 2018) (B). SXT, trimethoprim-sulfamethoxazole; nmMRSA, non-multidrug-resistant MRSA; mMRSA, multidrug-resistant MRSA; MSSA, methicillin-susceptible S. aureus.

Of 27,721 S. aureus isolates collected from 2011 to 2018, 2251 (8.1%) were reported as SXT resistant by Vitek 2. Reported SXT resistance rates fluctuated over time but did not substantially change between 2011 and 2018 (7% in 2011 versus 8% in 2018 [*P* = 0.12 for trend over time]) (Fig. 1A). However, SXT resistance due to mMRSA substantially decreased over time, with mMRSA isolates making up 81% of SXT-resistant isolates in 2011 versus 11% in 2018 (*P* < 0.001 for trend over time). In contrast, SXT resistance due to nmMRSA substantially increased over time (3% of isolates in 2011 versus 67% in 2018 [*P* < 0.001]), with nmMRSA replacing mMRSA as the dominant phenotype among SXT-resistant isolates. Among all 27,721 isolates, the proportion of S. aureus isolates that were MRSA (including nmMRSA) increased over time, making up 33% of isolates in 2011 versus 35% of isolates in 2018 (*P* = 0.001). Again, this increase was attributable to an increase in the frequency of nmMRSA isolates, which made up 25% of all isolates in 2011 but increased to account for 33% of S. aureus isolates by 2018 (*P* < 0.001 for trend over time) (Fig. 1B).

### Case-control study.

**FIG 2:**
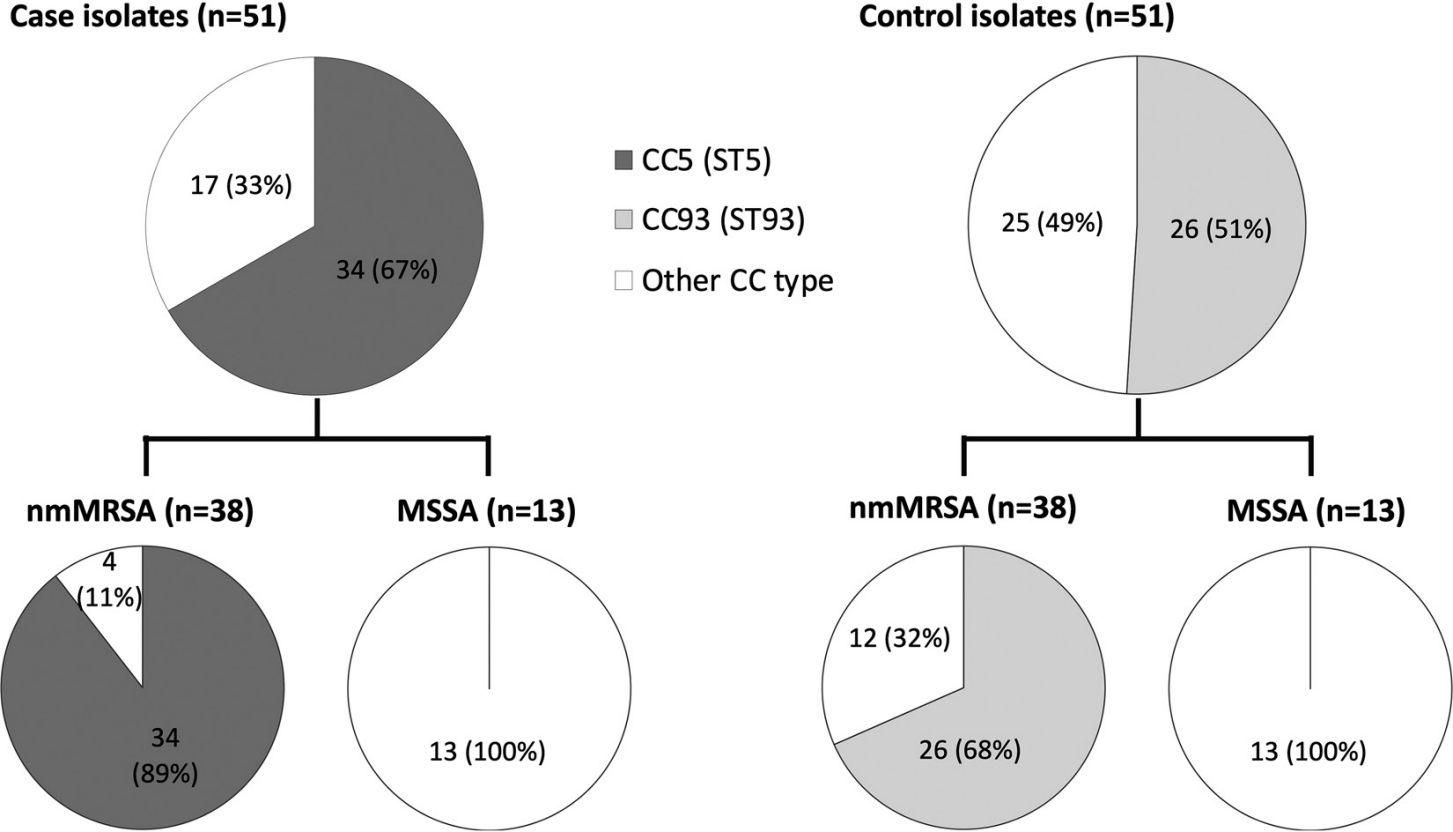
Genotyping results for S. aureus isolates among cases and controls. CC, clonal complex; ST, sequence type. Full genotyping results are available in Data Set S1.

We collected 51 case-control isolate pairs over a 6-month period in 2015. Demographic and clinical features of cases and controls were similar; most infections (88/102 [86%]) were SSTIs, and hospital admission (76/102 [75%]) and operative surgical drainage (45/102 [44%]) were common (Table 1). Of the 102 isolates collected, 76 were nmMRSA (74.5%) and 76 harbored the *lukSF-PV* genes encoding PVL (74.5%). Genotyping revealed clear differences among the nmMRSA isolates in the case and control groups (Fig. 2). Within the case isolates, 89% of the nmMRSA (34/38) belonged to a subset of clonal complex 5 (CC5) that includes ST5 (CC5/ST5), while CC93 predominated among nmMRSA in the control group (26/38 [68%]). All of the 34 CC5/ST5 case isolates were positive for *mecA*, and all but 1 were positive for PVL. All CC93 control isolates were positive for *mecA* and PVL. Genotyping results for all isolates are available in Data Set S1 in the supplemental material.

**TABLE 1:** Demographics and clinical features of infections caused by SXT-resistant (case) and SXT-susceptible (control) isolates^*a*^

Variable	Value for:		Unadjusted OR (95% CI)	*P* value
	Case isolates (*n* = 51)	Control isolates (*n* = 51)		
Demographics				
Age in yrs, median (range)	36 (0–89)	40 (0–96)		0.19
Male sex, no. (%)	27 (53)	27 (53)	1.00 (0.43–2.31)	1.00
Remote residence, no. (%)	22 (43)	23 (43)	0.93 (0.44–1.98)	0.85
Indigenous ethnicity, no. (%)	29 (57)	28 (55)	0.92 (0.42–2.02)	0.84
Diabetes, no. (%)	14 (27)	13 (25)	1.11 (0.45–2.73)	0.82
Scabies, no. (%)	6 (12)	6 (12)	1.00 (0.29–3.45)	1.00
Hazardous alcohol, no. (%)	8 (16)	16 (31)	0.43 (0.16–1.15)	0.08
Renal dialysis, no. (%)	2 (4)	2 (4)	1.00 (0.14–7.10)	1.00
Community onset, no. (%)	44 (86)	43 (84)	1.17 (0.39–3.47)	0.78
ICU admission, no. (%)	0 (0)	3 (6)		
Hospital admission, no. (%)	34 (67)	42 (82)	0.47 (0.19–1.14)	0.10
SXT exposure,^*d*^ no. (%)	3 (6)	1 (2)	3.46 (0.31–28.84)	0.34
MRSA in last 12 mo, no. (%)	5 (10)	9 (18)	0.55 (0.19–1.66)	0.29
Clinical presentation				
SSTI, no. (%)	44 (86)	44 (86)	1.00 (0.35–2.85)	1.00
Abscess	27 (53)	23 (45)	1.36 (0.63–2.97)	0.44
Wound infection	11 (22)	16 (31)	0.62 (0.26–1.48)	0.27
Cellulitis	6 (12)	5 (10)	1.20 (0.37–3.93)	0.76
Colonization, no. (%)	3 (6)	5 (10)	0.60 (0.14–2.51)	0.48
Other (bone and joint, line-related, pleuropulmonary infections), no. (%)	4 (8)	2 (4)	2.00 (0.37–10.92)	0.41
Any surgical management,^*b*^ no. (%)	28 (55)	35 (69)	0.50 (0.20–1.24)	0.13
Surgical management in operating theater,^*c*^ no. (%)	18 (35)	27 (53)	0.36 (0.13–0.99)	0.05
Death within 90 days, no. (%)	0 (0)	1 (2)		
Length of stay, median (range)	3 (0–61)	3 (0–469)		0.20
Microbiological and genotyping results, no. (%)				
MSSA	13 (25)	13 (25)		
nmMRSA	38 (75)	38 (75)		
Panton-Valentine leukocidin positivity	44 (86)	32 (63)		
MecA positivity	39 (77)	38 (75)		

a OR, odds ratio; CI, confidence interval; ICU, intensive care unit; SXT, trimethoprim-sulfamethoxazole; MRSA, methicillin-resistant Staphylococcus aureus; SSTI, skin and soft tissue infection; MSSA, methicillin-susceptible S. aureus; nmMRSA, non-multidrug-resistant methicillin-resistant S. aureus.

b Any surgical management of infective focus (e.g., incision and drainage or debridement), including procedures performed under local anesthetic in the emergency department or ward setting.

c Surgical management of infective focus in the operating theater.

d Completed or partially completed treatment course of SXT within the last 6 months as documented in the RDH electronic prescription database.

10.1128/mSphere.00651-20.1 DATA SET S1 Clinical and genotyping information for case and control isolates. Download Data Set S1, XLSX file, 0.02 MB. Copyright © 2021 McGuinness et al. 2021 McGuinness et al. https://creativecommons.org/licenses/by/4.0/ This content is distributed under the terms of the Creative Commons Attribution 4.0 International license.

We compared the epidemiological and clinical features of CC5 (*n* = 34) and CC93 (*n* = 26) isolates (Table 2). Most CC5 infections were community onset (30/34 [88%]), as were CC93 infections (22/26 [85%] [*P* = 0.72]). Most CC5 and CC93 isolates were recovered from patients living in remote communities (20/24 [59%] and 11/26 [46%], respectively [*P* = 0.30]) and from Indigenous patients (25/34 [74%] and 15/26 [58%], respectively [*P* = 0.27]). Clinical features were similar, with SSTIs predominating (88% in both groups). Abscess was the most common SSTI presentation, seen in 16/34 (47%) of those with CC5 isolates and 15/26 (58%) of those with CC93 isolates (*P* = 0.45). The majority of surgical procedures were for incision and drainage of abscesses and/or debridement of wounds. Patients with CC93 isolates were more likely to require surgical management in an operating theater (17/26 [65%]) than patients with CC5 isolates (12/34 [35%] [*P* = 0.02]). Patients with CC93 isolates were also more likely to be admitted to the hospital (24/26 [96%]) than those with CC5 isolates (22/34 [65%], *P* = 0.02), but hospital lengths of stay were similar in the two groups. Three patients with CC93 isolates (12%) were admitted to the intensive care unit (ICU), compared to no patients (0%) with CC5 isolates (*P* = 0.08), but two of the three admissions were for unrelated reasons (both cases of head trauma with either subarachnoid or subdural hemorrhage). Two patients with CC5 isolates had received treatment courses of SXT within the 6 months prior to S. aureus isolation, compared to one patient with a CC93 isolate. Readmission rates were low, and only one patient died within 90 days of S. aureus isolation; this was a 62-year-old man who was admitted following head trauma and died within 7 days of admission because of a subarachnoid hemorrhage. S. aureus was isolated from sputum and considered a colonizing organism.

**TABLE 2:** Demographics and clinical features of infections caused by CC5 (including ST5) versus CC93 isolates

Variable	Value for:		*P* value
	CC5 isolates (*n* = 34)	CC93 isolates (*n* = 26)	
Demographics			
Age in yrs, median (range)	35.5 (0–78)	41 (0–63)	0.23
Male sex, no. (%)	16 (47)	16 (62)	0.31
Remote residence, no. (%)	20 (59)	11 (42)	0.30
Indigenous ethnicity, no. (%)	25 (74)	15 (58)	0.27
Diabetes, no. (%)	12 (35)	6 (23)	0.40
Scabies, no. (%)	5 (15)	4 (15)	1.00
Hazardous alcohol, no. (%)	7 (21)	8 (31)	0.39
Renal dialysis, no. (%)	2 (6)	2 (8)	1.00
Community onset, no. (%)	30 (88)	22 (85)	0.72
ICU admission, no. (%)	0 (0)	3 (12)	0.08
Hospital admission, no. (%)	22 (65)	24 (92)	0.02
SXT exposure,^*c*^ no. (%)	2 (6)	1 (4)	1.00
MRSA in last 12 mo, no. (%)	4 (12)	3 (12)	1.00
Clinical presentation and outcome			
SSTI, no. (%)	30 (88)	23 (88)	1.00
Abscess	16 (47)	15 (58)	0.45
Superficial wound infection or impetigo	9 (26)	5 (19)	0.56
Cellulitis	5 (15)	3 (12)	1.00
Colonization, no. (%)	2 (6)	2 (8)	1.00
Other (bone and joint, line-related, pleuropulmonary infections), no. (%)	2 (6)	1 (4)	1.00
Any surgical management, no. (%)^*a*^	19 (56)	21 (81)	0.06
Surgical management in operating theater, no. (%)^*b*^	12 (35)	17 (65)	0.02
Death within 90 days, no. (%)	0 (0)	1 (4)	0.43
Length of stay, median (range)	3.4 (0–66)	5 (0–61)	0.44
Genotyping results			
Panton-Valentine leukocidin positivity, no. (%)	33 (97)	26 (100)	1.00
MecA positivity, no. (%)	34 (100)	26 (100)	

a Any surgical management of infective focus (e.g., incision and drainage or debridement), including procedures performed under local anesthetic in the emergency department or ward setting.

b Surgical management of infective focus in the operating theater.

c Completed or partially completed treatment course of SXT within the last 6 months as documented in the RDH electronic prescription database.

**FIG 3:**
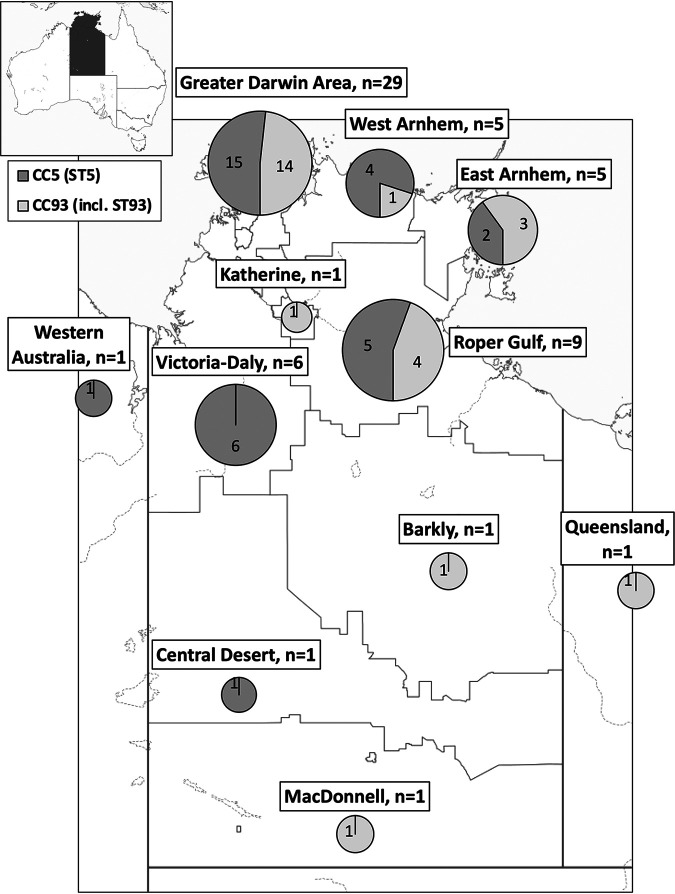
Map of distribution of ST5 and CC93 MRSA isolates. The greater Darwin area includes Darwin City, Palmerston City, and Litchfield Council areas, Belyuen and Coomalie Community Council areas, and the Tiwi Islands.

The geographical distribution of ST5 and CC93 MRSA isolates was mapped according to residential location at time of admission (Fig. 3).

**FIG 4:**
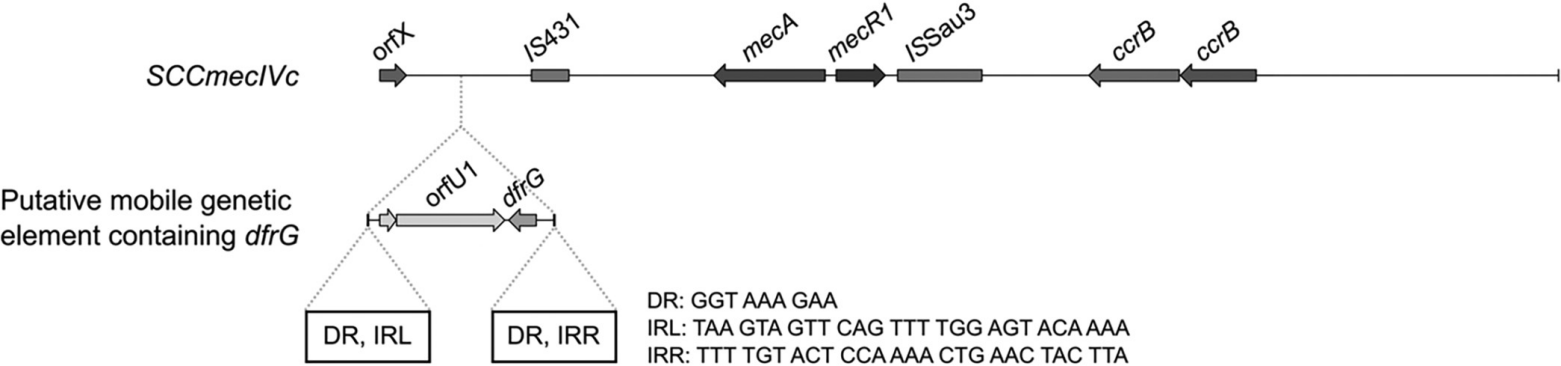
Structure of SCC*mec* IVo. SCC*mec* IVo is defined as SCC*mec* IVc harboring a putative mobile genetic element containing *dfrG* and two additional open reading frames (ORFU1 and ORFU2) encoding hypothetical proteins (18), inserted upstream of IS*431*. The direct repeats (DR) and left and right inverted repeats (IRL and IRR, respectively) of the putative *dfrG* mobile genetic element are indicated.

**FIG 5:**
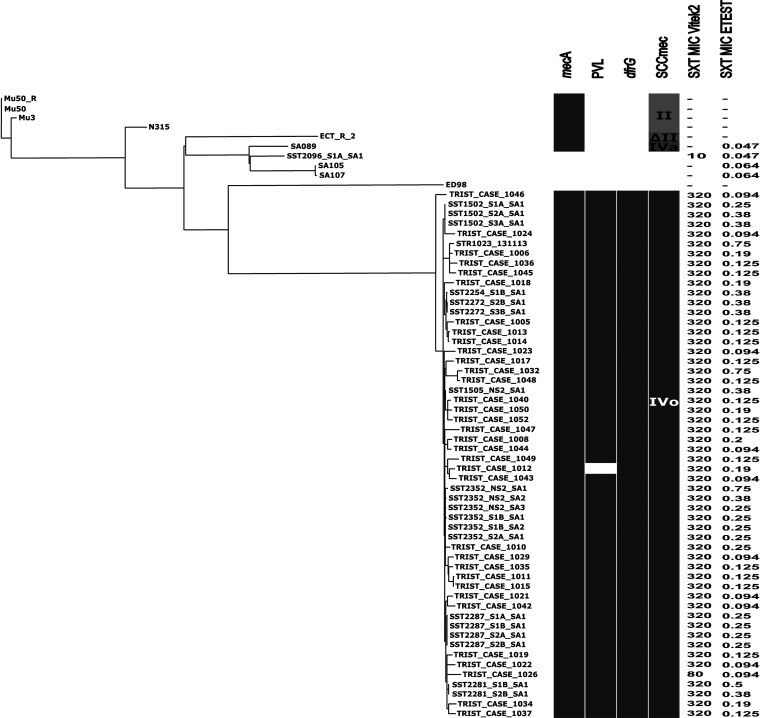
Phylogenomic analysis of ST5 isolates. Shown is a maximum-parsimony tree rooted on Mu50 of ST5 genome sequences based on 2,148 biallelic single nucleotide polymorphisms located in regions orthologous to all genomes (consistency index = 0.9581). Genome sequences included were those of 34 ST5 isolates from this study (indicated with the prefix “CASE_”), 24 ST5 isolates from a recent study of trimethoprim resistance in the same region (BioProject no. PRJNA312422) (16), and 5 publicly available ST5 isolates (ED98 [GenBank accession no. [NC_013450], ECT-R 2 [NC_017343], N315 [NC_002745], Mu3 [NC_009782], and Mu50 (NCBI reference sequence NC_002758.2; GenBank accession no. [NC_002758]). Synthetic short-read data (designated Mu50) generated from the Mu50 reference genome assembly (designated Mu50_R) were also included as an internal control for the alignment. The tree is available in Newick format in Text S1. Gray blocks to the right indicate the presence of *mecA*, *lukF/S* (PVL), *dfrG*, and SCC*mec*. Vitek 2 SXT MICs are reported as the sum of the trimethoprim and sulfamethoxazole MICs which are present in a ratio of 1:19, with a resistance breakpoint of ≥80 mg/liter (29). Etest SXT MICs are reported as the trimethoprim MIC, with a resistance breakpoint of >4 μg/ml (38). Scale represents 50 SNPs.

We conducted whole-genome sequence analysis of CC5/ST5 isolates. Genomic analysis demonstrated that all 34 CC5 isolates were ST5 and possessed SCC*mec* Ivo (16), which is closely related to SCC*mec* IVc in S. aureus strain TCH60. One hundred percent of the TCH60 SCC*mec* IVc can be aligned with SCC*mec* IVo, with a nucleotide identity of >98%. SCC*mec* IVo differs from the TCH60 SCC*mec* IVc by the insertion of a putative *dfrG* mobile genetic element upstream of IS431 (Fig. 4). This putative insertion element of 3,276 bp encompasses *dfrG* and two other open reading frames (ORFs) and is 100% identical to a sequence recovered from a clone in a genomic library of trimethoprim-resistant S. aureus isolate CM.S2 (GenBank accession no. AB205645.1) (18). Based on sequence identity, it was proposed that the CM-S2 *dfrG* may have been acquired by insertion sequence-mediated recombination from Enterococcus faecium (18). A search for potential markers of resistance to sulfamethoxazole across the genome demonstrated the absence of acquired sulfonamide resistance genes *sulI*, *sulII*, and *sulIII*. No nonsynonymous mutations in the chromosomal dihydropteroate synthase gene *folP* were observed in a comparison with an SXT-susceptible isolate (SST2096_S1A_SA1) (16). A maximum-parsimony phylogenomic tree was generated from 2,418 biallelic orthologous single nucleotide polymorphisms (SNPs) identified by the alignment of the ST5 isolates to Mu50 (NCBI reference sequence NC_002758.2 [GenBank accession no. NC_002758]). Isolates from a previous study which reported a circulating trimethoprim-resistant ST5 clone harboring SCC*mec* IVo from a similar region of Australia were also included for comparison; these isolates were collected between 2011 and 2013 (16). The ST5 isolates recovered in this study clustered closely with the trimethoprim-resistant ST5 isolates from the previous study, with a maximum of 65 SNPs separating any two isolates in this clade (Fig. 5). Resampling of the tree demonstrated 100% support for the major branches, including the clade clustering the trimethoprim-resistant ST5 isolates. Consistent with the previous report, the ST5 isolates recovered in this study were SXT susceptible by Etest, despite being reported as SXT resistant by Vitek 2 (Fig. 5).

10.1128/mSphere.00651-20.2 TEXT S1 Maximum-parsimony tree Newick format. Download TEXT S1, TXT file, 0.01 MB. Copyright © 2021 McGuinness et al. 2021 McGuinness et al. https://creativecommons.org/licenses/by/4.0/ This content is distributed under the terms of the Creative Commons Attribution 4.0 International license.

## DISCUSSION

Between 2011 and 2018, rates of Vitek 2 reporting of SXT resistance among S. aureus isolates were relatively stable; however, nmMRSA S. aureus strains almost completely replaced mMRSA strains as the predominant MRSA phenotype among SXT-resistant isolates. This phenomenon was driven by the emergence of a single ST5-MRSA-SCC*mec* IVo clone which possesses the trimethoprim resistance gene *dfrG* but does not harbor any acquired genes or mutations that potentially confer sulfamethoxazole resistance. Whole-genome sequence analysis demonstrated that ST5-MRSA-SCC*mec* IVo is causing a clonal outbreak across a large geographical region. Although additional phenotypic testing suggests *in vitro* susceptibility to trimethoprim-sulfamethoxazole, it is unclear at this stage whether the presence of *dfrG* within SCC*mec* IVo provides a selective advantage at the population level. This ST5-MRSA-SCC*mec* IVo clone is now the second most common community-associated MRSA strain in parts of Australia and causes a spectrum of clinical disease similar to that caused by the virulent ST93-MRSA lineage. Globally, *dfrG* is the most common trimethoprim resistance gene found in S. aureus, and it is typically located in mobile genetic elements (such as SCC*mec*) which can be transferred between bacteria (19, 20).

Discrepant SXT resistance reporting between Vitek 2 and Etest has recently been reported for S. aureus isolates from a previous study from our region (16). In that study, the discrepancy between Vitek 2 and Etest results was replicated in a second reference laboratory, with SXT susceptibility confirmed using Sensititre broth microdilution. The discovery of discrepant SXT susceptibility testing is concerning, as laboratories routinely reporting antimicrobial susceptibility for S. aureus using Vitek 2 results may be overcalling SXT resistance and this may translate to unnecessary restriction of effective oral treatment options for patients with S. aureus infections. Discrepancies in results by different testing methodologies are well recognized in laboratory practice. In light of the findings presented here and in a previous publication (16), the Royal Darwin Hospital (RDH) laboratory has made SXT Etest available on clinical request. Similarly, the major pathology provider for community clinics in the Northern Territory has amended protocols to include performing disc diffusion testing on all S. aureus isolates that test SXT resistant by Vitek 2. These are then reported as susceptible if found to be so on disc diffusion.

The majority of ST5 isolates investigated in our study were imported from the community into the hospital, and the infections typically occurred in Indigenous patients from remote communities. The disease caused by this clone is similar to that caused by other virulent PVL^+^ MRSA clones, such as ST93 (7, 21). Mapping of the location of disease onset (Fig. 3) suggests a greater relative prevalence of ST5 in the western regions of NT bordering WA than in the east of the NT. This is consistent with the reported emergence of a PVL^+^ ST5 clone (ST5-IV [2B]/WA 121) in northern WA, primarily among young Indigenous patients (17). The spread of ST93-MRSA from Indigenous communities in northwestern Australia to major population centers on the Australian east coast and subsequently overseas should serve as a salutary warning (8). Prospective surveillance of this ST5 lineage and appropriate community-based antimicrobial stewardship and infection control strategies will likely be important in containing its spread.

Other studies have shown that ST93 isolates are frequently methicillin-susceptible S. aureus (MSSA), whereas in this study all CC93 isolates were MRSA. This seeming inconsistency may be a consequence of the study design, in which cases and controls were matched with respect to MRSA/MSSA status, allowing MSSA CC93 to remain undetected in the very shallow sampling of the large number of diverse SXT-susceptible MSSA organisms isolated at RDH during the study period.

SXT use in northern Australia is almost certainly increasing, particularly in light of recent guidelines recommending the use of SXT for both impetigo and SSTIs (11, 14, 15). However, the role of increasing SXT prescriptions in the emergence and spread of either trimethoprim-resistant or SXT-resistant S. aureus is currently unclear. A time series analysis conducted in the United States did not demonstrate significant changes in SXT susceptibility rates despite increasing SXT prescriptions over time (9); however, these results may not be directly applicable to our setting, as the global epidemiology of S. aureus is heterogeneous and there are important geographical differences in predominant clones between the United States and Australia (5). Notably, SXT resistance is frequent in Africa, where SXT is commonly prescribed and SXT prophylaxis is used in HIV-infected individuals (22–25).

It is presently unclear whether the use of SXT will select SXT or trimethoprim resistance, and work is needed to elucidate this. In addition, further investigation into the role of increasing SXT prescriptions in SXT and trimethoprim resistance rates is required. Pilot surveillance of antimicrobial resistance rates and patterns and use of antimicrobial agents has commenced across the north of remote Australia to develop robust mechanisms for such surveillance (26). Targeted antimicrobial stewardship is a priority in the context of SSTIs dominated by nmMRSA to preserve effective oral antibiotic treatments. Although our prospective study is restricted to a hospital setting and limited by small numbers, it contributes an important understanding to the rapidly changing epidemiology of S. aureus in northern Australia, with implications for community-based treatment algorithms for SSTIs.

Our findings highlight the growing importance of genomic analysis in the investigation of emerging resistance trends. The absence of known acquired genes or mutations potentially conferring sulfamethoxazole resistance in genomic analysis of ST5 isolates prompted further phenotypic resistance testing. The discrepancy between Vitek 2 and SXT Etest results is consistent with that reported previously (16) and with the genomic analyses. The underlying reasons for the discrepancy between the Vitek 2 AST-P612 card and phenotypic Etest are not clear, and the issue was referred to bioMerieux for internal assessment. We note that when reference methods are compared there can be major variations in results between methods (27). Communications with bioMerieux indicate that although the discrepancy was confirmed when comparing the AST-P612 card with agar dilution as the reference, the discrepancy was not apparent when comparing the updated AST-P656 card with broth microdilution as the reference. As many laboratories are moving to the AST-P656 card, bioMerieux has stated that additional phenotypic testing is not required.

In conclusion, the almost complete replacement of mMRSA strains by nmMRSA strains as the predominant MRSA phenotype among Vitek 2-reported SXT-resistant isolates appears to be driven by the emergence of a trimethoprim-resistant ST5-MRSA-SCC*mec* IVo clone that is SXT susceptible by Etest. This clone, which has already been shown to be causing infections in the community setting (12), is now causing infections in patients presenting to hospitals and appears to cause clinical disease similar to that caused by the virulent ST93-MRSA-SCC*mec* IVa clone. In the context of increasing nmMRSA rates, empirical therapy for severe staphylococcal infections in our setting should clearly include non-beta-lactam agents that target MRSA. To date, SXT has been the preferred oral choice or step-down from intravenous therapy, but misleading SXT resistance reporting may deter clinicians from this option and lead to unnecessary restriction of effective oral treatment options for patients with S. aureus infections. Monitoring of clinical responses to treatment with SXT is recommended given some ongoing uncertainty regarding phenotypic SXT susceptibility testing.

## MATERIALS AND METHODS

### Design and setting.

The Royal Darwin Hospital (RDH) is a 350-bed tertiary referral center that serves a population of 170,000 over an area of approximately 500,000 km^2^ (28). RDH is the only tertiary referral hospital in the NT, a vast, sparsely populated territory of Australia that lies between the states of Queensland to the east and WA to the west. Indigenous Australians make up 27% of the regional population but comprise >50% of the RDH inpatient and emergency department population (28).

Using the RDH microbiology database, we collated antimicrobial susceptibility data for clinical S. aureus isolates from 2011 to 2018. Additionally, we conducted a prospective case-control study from March to August of 2015 that compared infections determined by Vitek 2 as SXT-resistant (case) and SXT-susceptible (control) S. aureus isolates. We identified S. aureus isolates using standard methods and performed antimicrobial susceptibility testing on the Vitek 2 platform (bioMerieux, France) using CLSI breakpoints (the CLSI SXT resistance breakpoint is ≥4/76 μg/ml) (29). Case isolates were consecutive S. aureus isolates resistant to SXT by Vitek 2. We matched case isolates 1:1 to the next eligible SXT-susceptible isolate (control) with the same oxacillin susceptibility profile (e.g., SXT-resistant MRSA matched to next SXT-susceptible MRSA). We defined isolates susceptible to oxacillin as methicillin-susceptible S. aureus (MSSA); isolates resistant to oxacillin and to <3 additional non-beta-lactam antibiotics were defined as nmMRSA (21). In our setting, the majority of isolates with a multidrug-resistant phenotype (mMRSA, those resistant to oxacillin and ≥3 additional non-beta-lactam agents) have previously been noted to be typically ST239, a hospital-adapted clone present in Australia for >20 years that is predictably resistant to SXT (30). As our interest was in emerging clones among MSSA and nmMRSA isolates, we excluded mMRSA isolates from the case-control study.

### Genotyping and genomic analysis.

Case and control isolates were genotyped using a previously described high-resolution melting technique (31), and PCR assays to detect the *nuc*, *mecA*, and *lukSF-PV* genes were performed (32). All isolates identified as belonging to clonal complex 5 (CC5), including ST5, were whole-genome sequenced on the Illumina HiSeq 2500 platform (Australian Genome Research Facility, Melbourne, Australia). Sequence quality was assessed using FastQC. Twelve additional isolates with poor or ambiguous genotyping results were also genome sequenced and subjected to *in silico* multilocus sequence typing (MLST). None of these isolates were determined to be ST5 isolates (Data Set S1), so no further genomic analysis was conducted. *In silico* MLST and identification of *mecA*, *lukSF-PV*, SCC*mec* IVo, and genes known to be associated with SXT resistance were performed using ARIBA v2.9.4 (33). To facilitate this, manually curated databases were created (*mecA*, *mecR1*, *mecI* [Mu50 genome, NCBI reference sequence NC_002758.2; GenBank accession no. NC_002758], *lukSF-PV* [TCH60 genome; GenBank accession no. CP002110.1], SCC*mec* IVo, *folP* [SST2096 genome; BioSample no. SAMN07460127], *sulI* [GenBank accession no. AF071413], *sulII* [GenBank accession no. EU360945], *sulIII* [GenBank accession no. HQ875016], *dfrG* [GenBank accession no. AB205645], and S. aureus PubMLST database [downloaded 7 August 2018]). Comparison of the SCC*mec* IVo region with the SCC*mec* IVc region of isolate TCH60 (GenBank accession no. CP002110.1) was performed using BLASTn. Short-read data of the 34 ST5 isolates from this study were aligned with Mu50 (NCBI reference sequence NC_002758.2; GenBank accession no. NC_002758) (34) to identify core single nucleotide polymorphisms (SNPs) and insertion/deletions (indels) using SPANDx v3.2.1 (35). Short-read data of 24 ST5 isolates from a recent study of trimethoprim resistance in the same region (BioProject accession PRJNA312422) (16) and synthetic short-read data generated using ART (36) for four additional publicly available ST5 isolates (ED98 [GenBank accession no. NC_013450], ECT-R 2 [NC_017343], N315 [NC_002745], and Mu3 [NC_009782]) were also included in this analysis. As an internal control of the alignment, synthetic short-read data (designated Mu50) generated from the Mu50 reference genome assembly (designated Mu50_R) was also included. A maximum-parsimony tree was built using PAUP v4.0a150 (37) and visualized using FigTree (http://tree.bio.ed.ac.uk/software/figtree/). Resampling of the tree with 1000 replicates was conducted.

### Additional antimicrobial sensitivity testing.

We determined SXT MICs using Etest (bioMerieux) for ST5 isolates. Isolates were cultured on horse blood agar (HBA; Oxoid, United Kingdom) for 18 h at 37°C with 5% CO_2_. Suspensions (0.5 McFarland in 0.85% physiological saline) were lawn inoculated onto Mueller-Hinton (MH) agar (Oxoid) and incubated at 35°C for 18 h. Etest SXT MICs were reported as the trimethoprim MICs in micrograms per milliliter as per manufacturer’s instructions (the EUCAST trimethoprim-sulfamethoxazole resistance breakpoint is >4/76 μg/ml) (38).

### Clinical data collection.

We extracted demographic and epidemiological data from RDH records. Extracted data included ethnicity, residential location at time of admission, underlying conditions (diabetes mellitus, hazardous alcohol consumption, intravenous drug use, renal replacement therapy, and scabies), admission and discharge details, clinical focus of infection, antibiotic therapy, and the number and type of surgical procedures. We also extracted information on health care-associated risk factors, including the presence of a permanent indwelling catheter or percutaneous medical device (e.g., Foley catheter), hospitalization, surgery, dialysis, isolation of MRSA within the past 12 months, and residence in a long-term-care facility. The following outcomes were recorded at days 7, 30, and 90 after isolation of S. aureus: hospitalization, readmission, and death. Colonization was defined as a positive culture for S. aureus at any site in a patient with no symptoms or biochemical indicators of infection and who did not require treatment. We defined recent SXT exposure as a completed or partially completed treatment course of SXT within the last 6 months as documented in the RDH electronic prescription database, which includes inpatient and outpatient prescription records. Hazardous alcohol consumption was defined as per “harmful” drinking in Australian guidelines (39). S. aureus infection was defined as community onset if isolation occurred within the first 48 h of presentation to the hospital. Using the residential location at time of admission, we mapped the distribution of CC5 and CC93 clones across a map of the NT.

### Statistical analysis.

We analyzed dichotomous variables using chi-squared or Fisher’s exact test and performed univariate conditional logistic regression to compare demographic and clinical features between case and control groups and looked for risk factors associated with Vitek 2-determined SXT resistance. We obtained a chi-square statistic for the trend of drug resistance rates over time. Two-sided *P* values of <0.05 were considered significant. Statistical analysis was performed using Stata 14 (StataCorp, TX).

### Ethics statement.

Ethics approval was granted by the Human Research Ethics Committee of the NT Department of Health and Menzies School of Health Research (HREC 2014-2176). The research was conducted in accordance with the Declaration of Helsinki and national and institutional standards.

### Data availability.

The short-read data for the genome sequenced ST5 isolates in this study have been deposited in the European Nucleotide Archive (ENA) at EMBL-EBI under accession number PRJEB40712).
